# New record of *Trissolcus solocis* (Hymenoptera: Scelionidae) parasitising *Halyomorpha halys* (Hemiptera: Pentatomidae) in the United States of America

**DOI:** 10.3897/BDJ.7.e30124

**Published:** 2019-02-19

**Authors:** Rammohan R Balusu, Ted E Cottrell, Elijah J Talamas, Michael D Toews, Brett R Blaauw, Ashfaq A Sial, David G Buntin, Edgar L Vinson, Henry Y Fadamiro, Glynn P Tillman

**Affiliations:** 1 Auburn University, Department of Entomology and Plant Pathology, Auburn, AL, United States of America Auburn University, Department of Entomology and Plant Pathology Auburn, AL United States of America; 2 United States Department of Agriculture, Agricultural Research Service, Southeastern Fruit & Nut Tree Research Laboratory, Byron, United States of America United States Department of Agriculture, Agricultural Research Service, Southeastern Fruit & Nut Tree Research Laboratory Byron United States of America; 3 USDA/SEL, Washington, DC, United States of America USDA/SEL Washington, DC United States of America; 4 Florida State Collection of Arthropods, Gainesville, United States of America Florida State Collection of Arthropods Gainesville United States of America; 5 Department of Entomology, University of Georgia, Tifton, GA, United States of America Department of Entomology, University of Georgia Tifton, GA United States of America; 6 Department of Entomology, University of Georgia, Athens, GA, United States of America Department of Entomology, University of Georgia Athens, GA United States of America; 7 Department of Entomology, University of Georgia, Athens, GA, United States of America Department of Entomology, University of Georgia Athens, GA United States of America; 8 Department of Entomology, University of Georgia, Griffin, GA, United States of America Department of Entomology, University of Georgia Griffin, GA United States of America; 9 Auburn University, Department of Horticulture, Chilton Research & Extension Center, Clanton, AL, United States of America Auburn University, Department of Horticulture, Chilton Research & Extension Center Clanton, AL United States of America; 10 United States Department of Agriculture, Agricultural Research Service, Crop Protection & Management Research Laboratory, Tifton, GA, United States of America United States Department of Agriculture, Agricultural Research Service, Crop Protection & Management Research Laboratory Tifton, GA United States of America

## Abstract

**Background:**

A parasitoid wasp, *Trissolcus solocis* Johnson, was recorded parasitising eggs of the invasive stink bug *Halyomorpha halys* (Stål), in the United States. This is the first record of this species parasitising eggs of *H. halys*.

**New information:**

First record of *Trissolcus solocis* parasitising *Halyomorpha halys* eggs in the United States and first record of *T. solocis* in Alabama.

## Introduction

The brown marmorated stink bug, *Halyomorpha halys* (Stål, 1855) (Hemiptera: Pentatomidae) (BMSB) is a native of China, Taiwan, Korea and Japan. Unfortunately, this invasive insect pest has spread to the United States (Lee et al. 2013), where it is both an urban nuisance pest (Inkley 2012) and a serious economic pest of orchard, field and vegetable crops (Leskey et al. 2012, Leskey et al. 2012, Rice et al. 2014). The first known *H. halys* populations in the United States were reported in 1996 from Allentown, PA (Hoebeke and Carter 2003). It has now been found in 44 states (StopBMSB 2018).

In the south-eastern U.S., populations of *H. halys* are continuing to expand into the Piedmont and Coastal Plains Regions of Georgia and Alabama. *Halyomorpha halys* was first detected in Alabama in 2010. One year later, urban pest management professionals began reporting overwintering brown marmorated stink bugs in homes in the metropolitan Atlanta area. Currently, the brown marmorated stink bug threatens peaches, plums, blueberries, apples, wine grapes, soybean, cotton, pecan and tomatoes in both States. The tree of heaven, *Ailanthus altissima* (Mill.) Swingle, a tree with seed pods that are a favourite non-crop food source for *H. halys*, also occurs in both States.

Presently, 18 species of hymenopteran endoparasitoids in the genera *Anastatus* Motchulsky (Eupelmidae), *Trissolcus* Ashmead, *Telenomus* Haliday and *Gryon* Haliday (Scelionidae) and *Ooencyrtus* Ashmead (Encyrtidae) have been reported to parasitise eggs of *H. halys* in the U.S. (Rice et al. 2014, Abram et al. 2014). As the impact of stink bug parasitoids on this pest was unknown in Georgia and Alabama, a survey to examine parasitism and species composition of parasitoids attacking sentinel egg masses of *H. halys* was conducted in 2017 in regions where populations of *H. halys* had become established.

## Materials and methods

Laboratory-reared *H. halys* egg masses were laid on knit cloth (97% cotton, 3% spandex) and were placed in containers. Egg masses (≤12 h old) were frozen and held at 20ºC for 1–4 d. On 20 September and 18 October, 30 egg masses were clipped or hung as sentinels on plants in cotton and the tree of heaven for 72 h. In the laboratory, the collected egg masses were held for emergence of adult parasitoids and emergent wasps were identified using the key of Talamas et al. (2015). Voucher specimens of parasitoids are deposited in the Florida State Collection of Arthropods, Gainesville, Florida.

All egg masses were dissected for dead immatures. Determination of *T. solocis* immature stages, mainly third instars, prepupae and pupae, were based on descriptions in Volkoff and Colazza 1992 and on dissections of *H. halys* eggs parasitised by another Trissolcus species, *T. basalis*, every 24 h from ovipostion to pupation.

## Taxon treatments

### Trissolcus solocis

Johnson, 1985

#### Materials

- **Type status:** Other material. **Occurrence:** recordedBy: R. Balusu; individualCount: 4; sex: male and female; lifeStage: adult; **Taxon:** scientificName: Trissolcus solocis; kingdom: Animalia; phylum: Arthropoda; class: Insecta; order: Hymenoptera; family: Scelionidae; genus: Trissolcus; specificEpithet: solocis; taxonRank: species; scientificNameAuthorship: Johnson, 1985; **Location:** continent: North America; country: United States of America; stateProvince: Alabama; municipality: Prattville; verbatimCoordinates: 32°42'86"N; 86°44'58"W; **Event:** samplingProtocol: sentinel egg; eventDate: 23/09/2017; habitat: cotton; **Record Level:** institutionCode: Florida State Collection of Arthropods; basisOfRecord: PreservedSpecimen
- **Type status:** Other material. **Occurrence:** recordedBy: R. Balusu; individualCount: 15; sex: male and female; lifeStage: adult; **Taxon:** scientificName: Trissolcus solocis; kingdom: Animalia; phylum: Arthropoda; class: Insecta; order: Hymenoptera; family: Scelionidae; genus: Trissolcus; specificEpithet: solocis; taxonRank: species; scientificNameAuthorship: Johnson, 1985; **Location:** continent: North America; country: United States of America; stateProvince: Alabama; municipality: Prattville; verbatimCoordinates: 32°42'86"N; 86°44'58"W; **Event:** samplingProtocol: sentinel egg; eventDate: 21/10/2017; habitat: sassafras; **Record Level:** institutionCode: Florida State Collection of Arthropods; basisOfRecord: PreservedSpecimen
- **Type status:** Other material. **Occurrence:** recordedBy: R. Balusu; individualCount: 3; sex: male and female; lifeStage: adult; **Taxon:** scientificName: Trissolcus solocis; kingdom: Animalia; phylum: Arthropoda; class: Insecta; order: Hymenoptera; family: Scelionidae; genus: Trissolcus; specificEpithet: solocis; taxonRank: species; scientificNameAuthorship: Johnson, 1985; **Location:** continent: North America; country: United States of America; stateProvince: Alabama; municipality: Prattville; verbatimCoordinates: 32°42'86"N; 86°44'58"W; **Event:** samplingProtocol: sentinel egg; eventDate: 21/10/2017; habitat: sassafras; **Record Level:** institutionCode: Florida State Collection of Arthropods; basisOfRecord: PreservedSpecimen
- **Type status:** Other material. **Occurrence:** recordedBy: R. Balusu; individualCount: 2; sex: female; lifeStage: adult; **Taxon:** scientificName: Trissolcus solocis; kingdom: Animalia; phylum: Arthropoda; class: Insecta; order: Hymenoptera; family: Scelionidae; genus: Trissolcus; specificEpithet: solocis; taxonRank: species; scientificNameAuthorship: Johnson, 1985; **Location:** continent: North America; country: United States of America; stateProvince: Alabama; municipality: Shorter; verbatimCoordinates: 32°49'86"N; 85°89'20"W; **Event:** samplingProtocol: sentinel egg; eventDate: 21/10/2017; habitat: cotton; **Record Level:** institutionCode: Florida State Collection of Arthropods; basisOfRecord: PreservedSpecimen
- **Type status:** Other material. **Occurrence:** recordedBy: R. Balusu; individualCount: 6; sex: male and female; lifeStage: adult; **Taxon:** scientificName: Trissolcus solocis; kingdom: Animalia; phylum: Arthropoda; class: Insecta; order: Hymenoptera; family: Scelionidae; genus: Trissolcus; specificEpithet: solocis; taxonRank: species; scientificNameAuthorship: Johnson, 1985; **Location:** continent: North America; country: United States of America; stateProvince: Alabama; municipality: Shorter; verbatimCoordinates: 32°49'86"N; 85°89'20"W; **Event:** samplingProtocol: sentinel egg; eventDate: 21/10/2017; habitat: cotton; **Record Level:** institutionCode: Florida State Collection of Arthropods; basisOfRecord: PreservedSpecimen

#### Diagnosis

**Figure 1.**
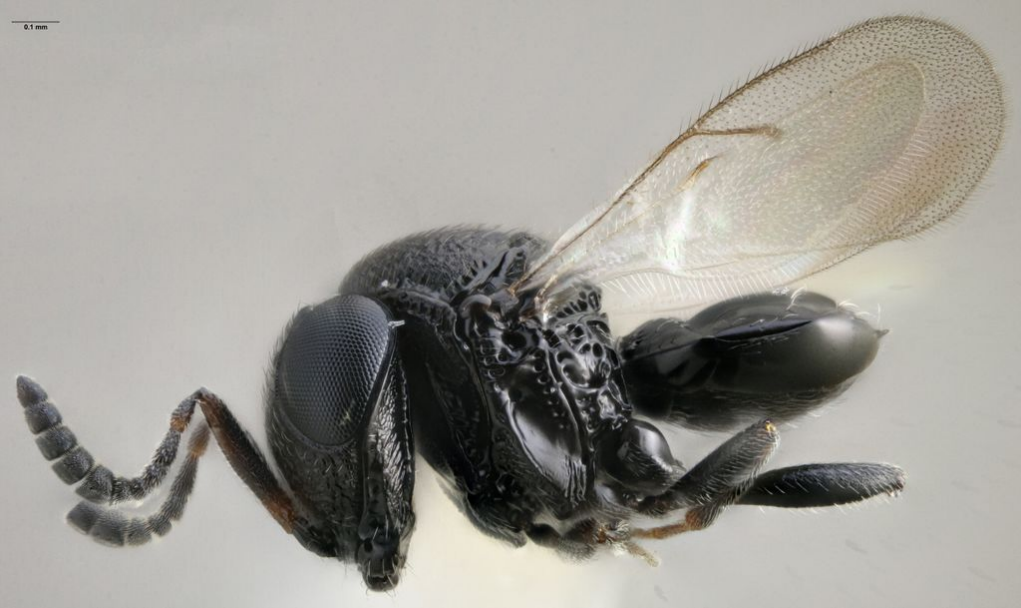
Female *Trissolcus solocis* whole body in lateral view.

Vertex without hyperoccipital carina; mesoscutellum coarsely rugose; clypeus with 6 setae; inner margin of eye with orbital furrow uniform in width, not expanded near malar sulcus; vertex sharply angled on to occiput; black radicle; well-defined paracoxal sulcus absent in the ventral half of the metapluron (Fig. 1) (Talamas et al. 2015).

#### Distribution

Distribution: *Trissolcus solocis* is known from Mexico and the south-eastern United States (http://hol.osu.edu/map-large.html?id=3311).

#### Biology

Additional host associations of *T. solocis* provided by are *Acrosternum marginatum* (Palisot), *Alcaeorrhynchus grandis* (Dallas) *Nezara viridula* (L.) and *Podisus maculiventris* (Say).

**Result**

Overall, the rate of parasitism in deployed sentinel egg masses was low, accounting for only 5.6%. The percent parasitism per egg mass was about 47.7%. Immature parasitoid mortality of 25.4% per egg mass was observed. Of the immature parasitoids that died, 81.8% were in larval instars, 15.2% were in pre-pupae and 3% were in the pupal stage. About 22.3% parasitoids per egg mass emerged as adults. The female-biased sex ratio of 2F:1M was observed in emerged parasitoids.

## Discussion

Multiple species of *Trissolcus* are known to oviposit into the eggs of *H. halys* despite a physiological inability to develop in them, creating an evolutionary trap (Abram et al. 2017, Abram et al. 2014, Haye et al. 2015). Two lines of evidence are consistent with *T. solocis* as a parasitoid that finds live BMSB eggs acceptable as a host, but not suitable. First, our records of *T. solocis* are only from frozen (dead) BMSB eggs. Second, *Trissolcus solocis* belongs to the *basalis* species group (sensu Johnson 1985, Talamas et al. 2017), whereas the species able to successfully parasitise BMSB in its native range belong to the *flavipes* group (sensu Talamas et al. 2017). In the absence of controlled experiments, we cannot draw conclusions from the present study beyond documentation of previously frozen BMSB eggs as an acceptable host for *T. solocis*.

## Supplementary Material

XML Treatment for Trissolcus solocis
